# An overall and dose-response meta-analysis of red blood cell distribution width and CVD outcomes

**DOI:** 10.1038/srep43420

**Published:** 2017-02-24

**Authors:** Haifeng Hou, Tao Sun, Cheng Li, Yuanmin Li, Zheng Guo, Wei Wang, Dong Li

**Affiliations:** 1 School of Public Health, Taishan Medical University, Taian, 271000, China; 2 School of Medical and Health Sciences, Edith Cowan University, Perth, Australia; 3 Ruijin Hospital, Shanghai Jiao Tong University, Shanghai, 200025, China; 4 Affiliated Hospital, Taishan Medical University, Taian, 271000, China

## Abstract

Red blood cell distribution width (RDW) is the coefficient of variation of red blood cell size, considered to be associated with cardiovascular disease (CVD). This study aimed to comprehensively synthesize previous studies on RDW and CVD outcomes through an overall and dose-response meta-analysis. PubMed, Embase and Web of Science were searched systematically for English and Chinese language publications up to November 30, 2015. We extracted data from publications matching our inclusion criteria for calculating pooled hazard ratio (HR), which was used to assess prognostic impact of RDW on CVD. Twenty-seven articles, consisting of 28 studies and 102,689 participants (mean age 63.9 years, 63,703 males/36,846 females, 2,140 gender-unmentioned subjects) were included in the present meta-analysis. The pooled HRs are 1.12 (95% CI = 1.09–1.15) for the association of all-cause mortality (ACM) per 1% increase of RDW, 1.12(95% CI = 1.08–1.17) for major adverse cardiac events (MACEs) per 1% increase of RDW. A dose-response curve relating RDW increase to its effect on CVD outcomes was established (*p*_curve_ < 0.001). For every 1-unit increase of RDW, there is an increased risk of occurrence of ACM (pooled HR = 1.03, 95% CI = 1.02–1.04) and MACEs (pooled HR = 1.04, 95% CI = 1.01–1.06). This study indicates RDW may be a prognostic indicator for CVD outcomes.

Red blood cell distribution width (RDW) is a quantitative parameter in routine hematologic analysis for representing the variation of red blood cell size. RDW is numerically expressed as the coefficient of variation for red blood cell size (equal to standard deviation divided by mean) and has been used to differentiate the causes of anemia for a long time^1^,^2^. Recently, the clinical significance of higher RDW has been considered in relation to cardiovascular disease (CVD), autoimmune disease and respiratory disease^3^,^4^,^5^,^6^. In these non-hematologic disorders, RDW is one of significant indicators of morbidity and mortality^7^,^8^. Clinical studies conducted among CVD patients have reported that RDW is a novel, independent predictor for all-cause mortality (ACM), and major adverse cardiac events (MACEs) as well^5^,^9^,^10^,^11^,^12^,^13^, which is the most important outcomes of CVD. In particular, MACEs include cardiovascular death, nonfatal myocardial infarction (MI) and heart failure (HF). Whereas the results are inconsistent and the underlying mechanism is unclear^14^,^15^. Even though some meta-analyses have combined the findings of RDW on predicting cardiovascular risk^14^,^15^, no dose-response meta-analysis has been performed^16^. Thus, we conducted the first overall and dose-response meta-analysis to offer new evidence on the association of RDW with CVD outcomes. The prognostic value of RDW for ACM and MACEs were then comprehensively reviewed among CVD (e.g. coronary artery disease, HF, MI) patients.

## Results

### Search results and study characteristics

**Figure 1:**
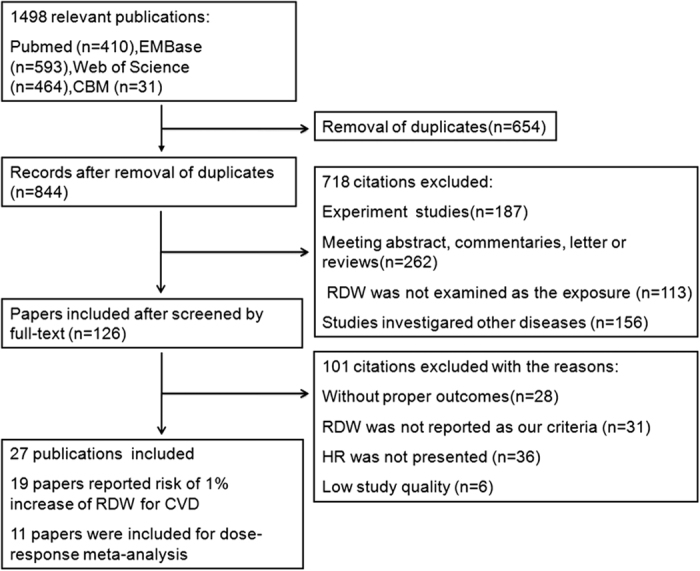
Flow chart of the study selection.

As shown in flowchart (Fig. 1), our literature search returned 1,498 publications. After screening the title and abstract of these articles, we kept 126 ones for full-text review. Consequently, this meta-analysis included 27 articles with 28 independent studies and 102,689 participants (mean age 63.9 years, 63,703 male/36,846 female, 2,140 gender-unmentioned subjects), the characteristics of which were provided in Table 1.

### Overall meta-analyses of prognostic value of RDW for ACM among CVD patients

**Figure 2:**
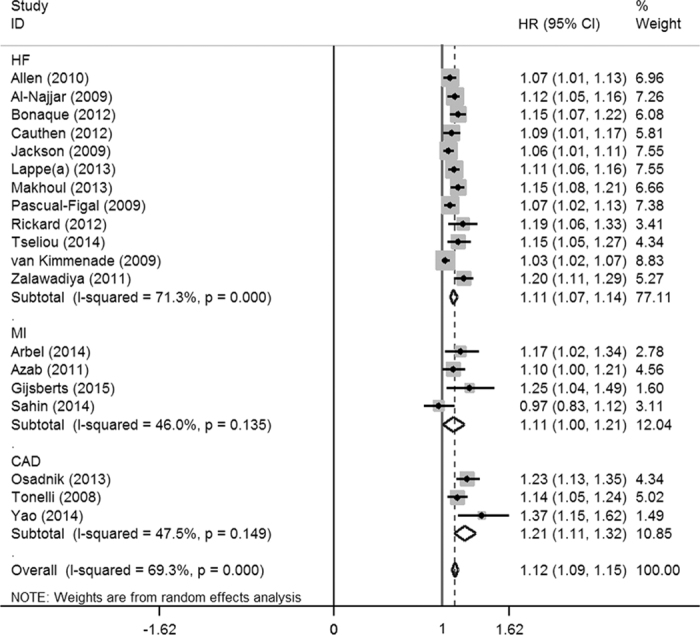
Forest plot of the pooled adjusted HR of per 1% RDW increase for the risk of all-cause mortality in overall meta-analysis. The size of each grey square is proportional to the study’s weight calculated in the meta-analysis. HR: hazard ratio; 95% CI: 95% confidence interval; HF: heart failure; MI: myocardial infarction; CAD: coronary artery disease.

As shown in Fig. 2 and Supplementary Table 3, 19 original studies^1^,^6^,^9^,^11^,^17^,^18^,^19^,^20^,^21^,^22^,^23^,^24^,^25^,^26^,^27^,^28^,^29^,^30^,^31^ reported the prognostic risk of per 1% increase of baseline RDW for all-cause mortality in CVD patients. After combining adjusted hazard ratios (HRs) and 95% confidence interval (CI) of the 19 studies, we found that the ACM risk of CVD patients significantly increased by 12% (pooled HR = 1.12, 95% CI = 1.09–1.15, *p* = 0.000) per 1% increase of RDW among CVD patients. The heterogeneity test showed that statistical significance existed across all the studies (I^2^ = 69.3%, Q = 58.55, *p* = 0.000). The subgroup analysis was conducted based on specific disease category. The pooled HRs (and 95% CIs) of ACM were 1.11(1.07–1.14), 1.11(1.00–1.21), 1.21(1.11–1.32) among HF, MI and CAD patients, respectively. In addition, the results of subgroup analyses stratified by subject ethnicity, study design and follow-up duration were presented in Supplementary Table 3.

### Overall meta-analyses of prognostic value of RDW for MACEs among CVD patients

**Figure 3:**
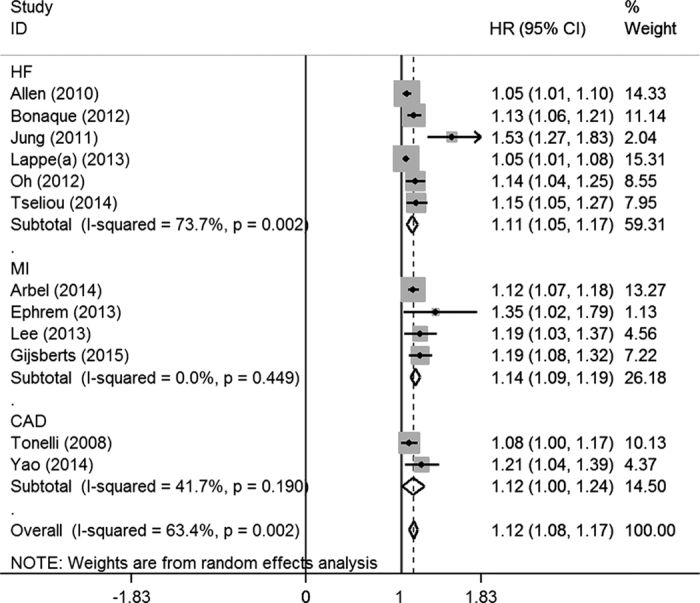
Forest plot of the pooled adjusted HR of per 1% RDW increase for the risk of MACEs in overall meta-analysis. The size of each grey square is proportional to the study’s weight calculated in the meta-analysis. HR: hazard ratio; 95% CI: 95% confidence interval; HF: heart failure; MI: myocardial infarction; CAD: coronary artery disease.

Eleven studies^5^,^17^,^20^,^22^,^26^,^28^,^29^,^31^,^32^,^33^,^34^ addressed the relationship between per 1% increase of RDW and MACEs. The pooled HR (1.12, 95% CI = 1.08–1.17, *p* = 0.000) indicated that increased RDW also facilitated MACEs among CVD populations (Fig. 3). The heterogeneity was significant across the 11 studies (I^2^ = 63.4%, Q = 30.09, *p* = 0.003). Then, we performed subgroup analyses based on disease classification, subject ethnicity, study design and follow-up duration. As shown in Supplementary Table 4, the pooled HRs (95% CIs) were 1.11(1.05–1.17) among HF patients, 1.14(1.08–1.21) among MI patients, and 1.12(1.08–1.17) among CAD patients, respectively.

### Dose-response meta-analysis on relationship between RDW and ACM among CVD patients

**Figure 4:**
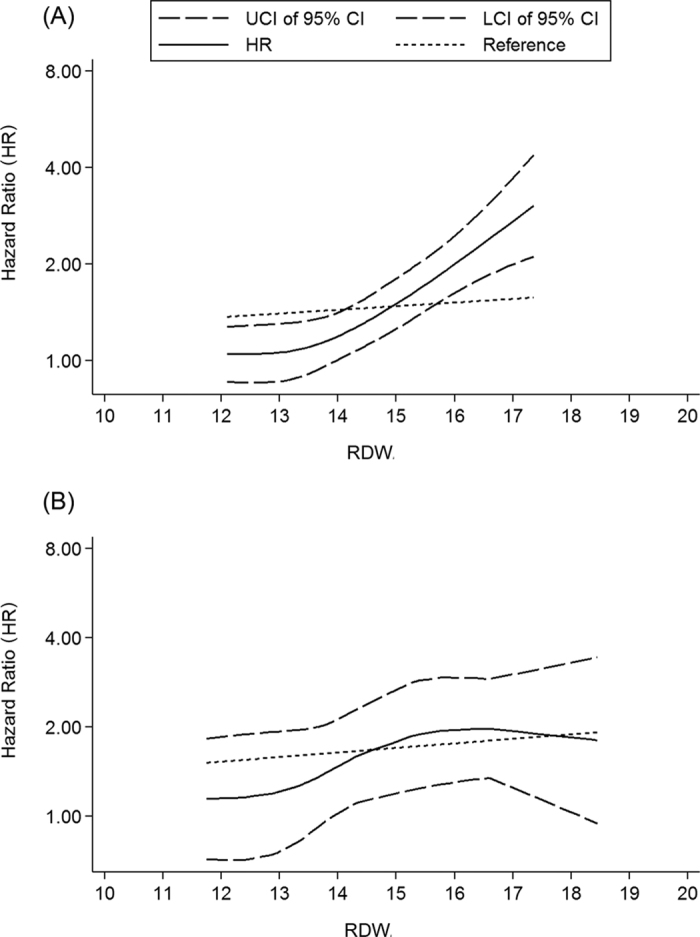
Dose-response relationship between RDW (per 1-unit increase) and CVD outcomes. (**A**) Relationship between RDW and all-cause mortality. (**B**) Relationship between RDW and MACEs. Dotted lines represent the 95% CI for the fitted trend. LCI: lower limit of confidence interval; UCI upper limit of confidence interval; 95% CI: 95% confidence interval; RDW: red blood cell distribution width.

Twelve original studies within 11 articles^2^,^8^,^20^,^23^,^27^,^28^,^35^,^36^,^37^,^38^,^39^ were included in the dose-response meta-analysis on prognostic value of RDW for all-cause mortality, in which the HRs of RDW classification (e.g. tertile, quartile or quintile) for CVD risk were published. As the curve showed (Fig. 4A), the dose-response relationship between RDW and ACM was significant (χ^2^ = 48.57, *p* = 0.000). The pooled HR was 1.03(95% CI = 1.02–1.04) for every 1-unit increase of RDW among CVD patients.

### Dose-response meta-analysis on relationship between RDW and MACEs among CVD patients

Five studies^2^,^12^,^20^,^28^,^40^ were combined in the dose-response meta-analysis of association between RDW level and MACEs. All studies provided the risks of RDW classification for MACEs. As shown in Fig. 4B, a significant curve of dose-response relationship existed (χ^2^ = 10.25, *p* = 0.001), with the pooled HR being 1.04 (95% CI = 1.01–1.06) for every 1-unit increase of RDW.

### Dose-response meta-analysis on relationship between RDW/Hb and outcomes of CVD

**Figure 5:**
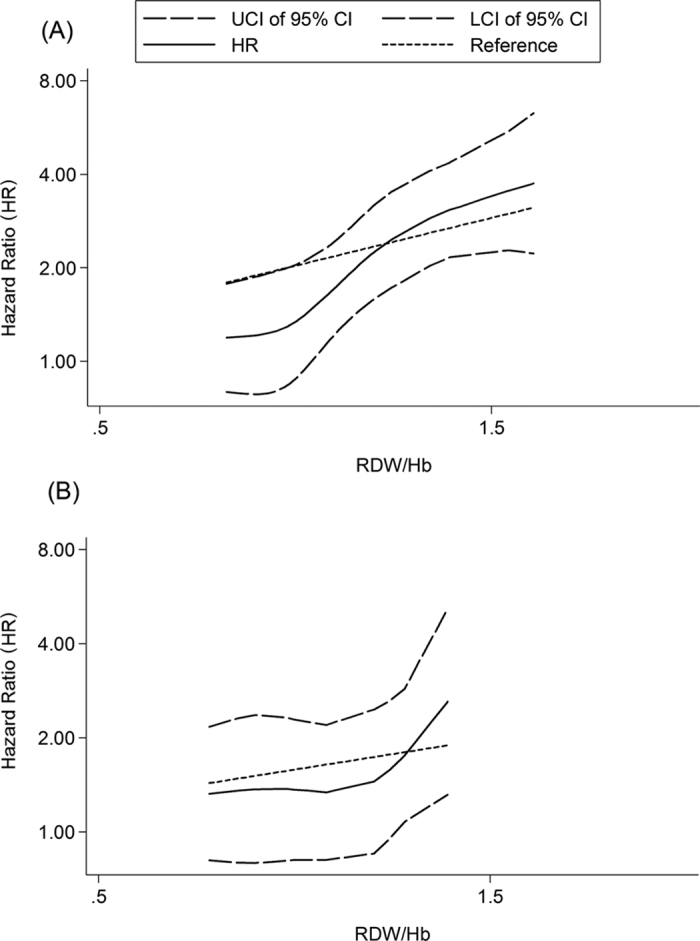
Dose-response relationship between RDW/Hb (per 1-unit increase) and adverse cardiovascular event risk. (**A**) Relationship between RDW and all-cause mortality. (**B**) Relationship between RDW and MACEs. Dotted lines represent the 95% CI for the fitted trend. LCI: lower limit of confidence interval; UCI upper limit of confidence interval; 95% CI: 95% confidence interval; RDW: red blood cell distribution width; Hb: hemoglobin.

In seven studies^12^,^20^,^23^,^28^,^36^,^37^,^41^ that reported mean hemoglobin(Hb) of subjects, the differences of Hb level between all RDW classifications were significant. To adjust the confounding of anemia status for CVD patients, we normalized RDW with Hb through calculating the ratio of RDW to Hb. Furthermore, we performed a dose-response meta-analysis to evaluate the prognostic value of RDW/Hb for ACM. As shown in Fig. 5A the curve of dose-response relationship between RDW and ACM was obvious (χ^2^ = 34.87, *p* = 0.000). The pooled HR was 2.03(95% CI = 1.60–2.57) for every 1-unit increase of the RDW/Hb ratio. For the relationship between RDW/Hb and MACEs, a significant curve of dose-response relationship exists (χ^2^ = 7.91, *p* = 0.048), with the pooled HR being 1.58(95% CI = 1.09–2.29) for every 1-unit increase of RDW/Hb (Fig. 5B).

### Sensitivity analysis

Anemia, resulting from percutaneous coronary intervention (PCI) in clinical settings, may be a confounder in this study. Therefore we conducted a sensitivity analysis by excluding the original studies of Poludasu *et al*.^38^ and Uyarel *et al*.^12^, as these CVD patients were recruited undergoing PCI. The results indicated that curve relationship between every 1-unit increase of RDW and adverse outcomes of CVD was significant, with a HR of 1.03(95% CI = 1.14–1.04, *p* = 0.000) for ACM and 1.04(95% CI = 1.01–1.06, *p* = 0.012) for MACEs (Supplementary Figures 1–2). Further sensitivity analysis was performed to detect the stability of this meta-analysis by removing each study sequentially. Consequently, no obvious change was generated for omission of each study except van Kimmenade’s research^6^. The detailed results of sensitivity analysis were described in Supplementary Figures 3–4.

### Publication bias

The funnel plot is used to explore potential publication bias of the current meta-analysis. Supplementary Figures 5–6 demonstrates that publication bias was statistically significant.

## Discussion

In this systematic review, we included 27 articles consisting of 28 original studies and 102,689 subjects. The pooled results demonstrated that baseline RDW level was remarkably associated with CVD outcomes. We found a 0.12-fold elevation for all-cause mortality risk in CVD patients per 1% increase of RDW. Increased RDW is also an effective predictor for MACEs with a HR of 1.12. The novel approach of dose-response meta-analysis identified the curve of dose-response relationship between RDW and CVD outcomes.

Red blood cell distribution width (RDW) is a parameter of the heterogeneity of circulating erythrocytes size, which was reported to be associated with CVD^1^,^2^,^6^,^12^,^28^,^37^,^38^. Although the mechanism of this association is not fully understood, RDW is considered to be an indicator of inflammation, and is implicated in several inflammatory markers, such as C-reactive protein (CRP), interleukin-6 (IL-6), and tumor necrosis factor-α (TNF-α)^19^,^42^. Inflammatory stress leads to dysfunctional bone marrow with ineffective production of red blood cells^18^, disturbs the red cell membrane and effectively causes the migration of reticulocytes into the peripheral circulation. This induces an increase in the proportion of immature RBCs in the circulation, resulting in higher RDW levels^42^. Moreover, inflammation up-regulates the expressions of complement protein receptors (C1qRs) and toll-like receptors (TLRs) in platelets. This contributes to platelet activation, accelerating the progression of inflammatory diseases, such as CVD^18^. It is clear that the inflammatory process is a principal pathophysiologic pathway in the development of CVD and CVD events^43^,^44^,^45^,^46^. In addition, an increase in RDW is related to increased oxidative damage in blood circulation, which associates with exacerbation of CVD^47^.

Hemodynamic status, reported by Salvagno *et al*., may play a role in association of RDW with CVD^48^. RBCs may become entrapped in atherosclerotic plaque by the occurrences of fibrous cap damage, thrombus formation or plaque hemorrhage resulting from injury of intraplaque microvessels. In these instances, RBCs accelerate atherogenesis locally in the damaged area. The high anisocytosis of RBCs, exhibited as increased RDW, causes decreased erythrocyte deformability. This may increase blood viscosity, disturb blood flow through the microcirculation, and promote the adverse consequences of a pre-existing vascular occlusion in CVD. Therefore, increased RWD levels may contribute to the identification of CVD patients who require more intensive therapy^18^.

Many clinical studies and several meta-analyses have been conducted to explore the application measures of the easily acquired routine blood index, and to study the prognostic significance of RDW among CAD or HF patients^14^,^15^. Whilst they have provided effective methods with valuable results, no authors conducted dose-response meta-analysis. Does–response meta-analysis is a new method of systematic review and meta-analysis for assessing the effect of continuous quantitative variables on diseases^16^. Compared to previous meta-analyses, our study found a dose-response relationship between RDW and CVD outcomes.

Although RDW is a recognized parameter associated with anemia, no study has provided an explicit baseline for anemia with exception to hemoglobin (Hb) levels in subjects. In the present study, we calculated the pooled HR, for which Hb and other confounding factors were adjusted with multiple statistical model. A recently publication verified that RDW was an independent risk factor related to mortality of post-PCI non-anemia patient^49^. This finding indicates that RDW is an independent predictor of CVD mortality^49^. To further adjust the effect of anemia on our results and explore the appropriate rheological role of related risk factors, we normalized RDW with Hb. We found that the ratio of RDW/Hb is significant for predicting CVD outcomes in a dose-response manner. This is the first report demonstrating the prognostic value of the normalized RDW (i.e. RDW/Hb) on CVD outcomes. As RBC count is also considered to be independently associated with CVD risk^50^, we suggest that other approaches of normalizing RDW, such as the ratio of RBC count, might be utilized in further relevant studies for the purpose of explaining rheological role of RDW on CVD outcomes.

Moreover, the treatment of PCI plays a crucial role in post-PCI anemia because of arterial vessel wall injury and further antiplatelet or antithrombotic medication during the PCI procedure. Post-PCI anemia is also reported to increase the risk of CVD in patients^51^. Further, our sensitivity analysis, excluding the original studies conducted among participants undergoing PCI, showed a significant dose-response relationship between RDW and CVD outcomes.

The present study has certain limitations. Firstly, the heterogeneity across individual studies was not avoided completely. Secondly, we did not conduct subgroup analyses based on gender and age stratifications because no original articles reported the detailed HRs stratified by age and gender. Thirdly, the potential publication bias might disturb the quality of meta-analysis. Publication bias is a disadvantage in systematic reviews and meta-analyses, which needs to be mitigated as much as possible at the onset of original studies. When researchers evaluate the risks of RDW and other variables on CVD outcomes, the COX proportional hazards regression model would be utilized to calculate the adjusted HRs, which then presented the independent risk degrees. However, the non-significant HRs were not included in the original publications. This is problematic for us to further systematic reviews and meta-analyses referring to these studies, as only the significant adjusted HRs were included in synthesized HRs. Undoubtedly, this induced a publication bias into our studies. In order to avoid this kind of bias in the future, the following methods should be implemented: (1) all results of statistical analysis should be reported, (2) when employing a stepwise method for COX regression analysis, the variables in the multivariate model equation and those not in the equation should be involved simultaneously; otherwise, the *enter* model should be applied to include each variable in multivariate statistical analysis.

In spite of the afore-mentioned limitations, this study presents new comprehensive evidence for the association between RDW and CVD outcomes. We might conclude that increased RDW is a prognostic indicator for CVD outcomes with a dose-response manner.

## Methods

This meta-analysis is conducted according to the published criteria of the Preferred Reporting Items for Systematic Reviews and Meta-Analyses (PRISMA)^14^,^15^. The PRISMA Checklist form is listed in Supplementary Table 1.

### Search strategy

We identified relevant studies on the association between RDW and adverse outcomes among CVD patients (i.e. coronary artery disease, MI, and HF). The search approach was performed to retrieve articles from PubMed, Embase and Web of Science databases published in English and Chinese up to November 30, 2015. The search strategy was designed with the following terms: “red cell distribution width” or “red blood cell distribution width” or “RDW”, “mortality”, “CVD events”, “cardiovascular disease” or “CVD”, “coronary artery disease” or “CAD”, “myocardial infarction” or “MI”, and “heart failure” or “HF”. Further manual collection of references attached on retrieved papers was performed to screen potential relevant studies.

### Selection criteria

We included studies that fulfilled the following inclusion criteria: (1) the baseline or admission serum RDW level was reported; (2) prospective study or retrospective study that evaluated the prognostic value of RDW for CVD patients; (3) one of the following outcomes was reported: all-cause mortality (ACM), fatal CVD events (cardiovascular death), non-fatal CVD events (e.g. MI, stroke, HF and readmission for CVD); (4) studies performed in participants aged ≥18 years. We excluded studies that matched any of the following exclusion criteria: (1) duplicated data; (2) researches based on animal or cell line design; (3) no full data can be obtained.

### Quality assessment and data extraction

Two authors independently reviewed relevant articles. The quality of each study was assessed with the scale for quality assessment (Supplementary Table 2) generated according to the PRISMA statement and the Meta-analysis of Observational Studies in Epidemiology (MOOSE) guidelines^15^. The studies labeled high quality with score ≥6 were included in the current meta-analysis. The third reviewer (D.L.) contributed to the resolution of inconsistent opinions.

The following data for eligible studies were extracted independently: year of publication, name of first author, duration of follow-up, location of study population, number of participants, characteristics of patients or controls, definition of outcome, adjusted HR for per 1% increase of RDW for CVD risk, adjusted HR for each RDW classification compared to reference level. We also checked the online Supplementary Data of published articles when necessary.

### Data synthesis and statistical analysis

The STATA 14.0 software (by Stata Corp, College Station, TX, USA) was utilized to analyze data. We combined HR values to identify the prognostic risk of per 1% increase of RDW for CVD based on the original studies reporting quantitative RDW levels. For other studies which addressed ordinal classification of RDW levels, the dose-response meta-analysis was performed to synthesize pooled HRs and the curve of dose-response relationship with the method of Greenland and Longnecker^16^. The number of cases, person-years or numbers of all participants are required for this methodology, and the HR (and 95% CI) for at least three RDW categories are required as well. For the studies that did not publish the number of cases or person-years in each RDW level, the data were calculated approximately from total number of cases, person-years and HR in this study. The I^2^ and Q-test was performed to detect the heterogeneity across included studies. The fixed effects model was used to combine the original data when the heterogeneity was not statistically significant. Otherwise, the random effects model analysis was used when heterogeneity was considered to be significant (*p* was < 0.10 and I^2^ was >50%). Furthermore, subgroup analyses were performed to explore the sources of heterogeneity according to study characteristics in terms of study design, type of disease, follow-up duration and ethnicity of subject. Sensitivity analyses were conducted to evaluate the stability of results in the current meta-analysis. Funnel plot analysis was implemented to detect potential publication bias^52^.

## Additional Information

**How to cite this article:** Hou, H. *et al*. An overall and dose-response meta-analysis of red blood cell distribution width and CVD outcomes. *Sci. Rep.* **7**, 43420; doi: 10.1038/srep43420 (2017).

**Publisher's note:** Springer Nature remains neutral with regard to jurisdictional claims in published maps and institutional affiliations.

## Supplementary Material

Supplementary Materials

## Figures and Tables

**Table 1:** Characteristics of included studies on association of RDW with outcomes of CAD.

Author	Year	Country	Disease	outcome	Sample size	Baseline	Follow up duration	Hazard Ratio (95% CI)
Allen LA	2010	USA	HF	ACM/MACE	1012	Age: 64 ± 14, 58% male	1.0 ± 0.3 y	HR (a) = 1.07 (1.01–1.13)*
								HR (m) = 1.05 (1.01–1.10)*
Al-Najjar Y	2009	UK	HF	ACM	1087	Age: 60 ± 78, 74.3% male	52 m	HR (a) = 1.12 (1.05–1.16)*
Anderson JL	2007	USA	CAD	ACM	29526	Age: 61.1 ± 14.7, 62% male	4.9 y	RDW ≤ 12.6, HR (a) = 1
								RDW = 12.7–13.2, HR (a) = 1.2 (0.8–1.8)
								RDW = 13.3–14.0, HR (a) = 1.3 (0.9–1.8)
								RDW ≥ 14.1, HR (a) = 1.8 (1.2–2.5)
Arbel Y (a)	2014	Israel	MI	ACM	535	Age: 60.5 ± 13.0, 80.1% male	5 y	HR (a) = 1.17 (1.025–1.34)*
Arbel Y (b)	2014	Israel	CAD	MACE	3222	Age: 65.6 ± ± 12.0, 72.7% male	415 d	HR (m) = 1.12 (1.07–1.18)*
Azab B	2011	USA	MI	ACM	619	Age: 64.1 ± 10.8, 69.7% male	4 y	HR (a) = 1.104 (1.004–1.213)*
Benedetto U	2013	Italy & UK	CAD	ACM	8340	Age: 66 ± 13, 85% male	4485 d	RDW ≤ 12.9, HR (a) = 1
								RDW = 12.9–13.4, HR (a) = 1.47 (1.09–3.78)
								RDW = 13.4–14.0, HR (a) = 2.47 (1.31–4.23)
								RDW > 14, HR (a) = 3.44 (2.5–4.7)
Bonaque JC	2012	Spain	HF	ACM/MACE	698	Age: 71 (62–77), 63% male	2.5 y	HR (a) = 1.15 (1.07–1.22)*
								HR (m) = 1.13 (1.06–1.21)*
								RDW<13.8, HR (a) = 1
								RDW = 13.8–14.8, HR (a) = 1.31 (0.83–2.06)
								RDW = 14.8–16, HR (a) = 2.05 (1.33–3.16)
								RDW > 16 = 3, HR (a) = 1.47 (2.29–3.16)
								RDW<13.8, HR (m) = 1
								RDW = 13.8–14.8,
								HR (m) = 1.32 (0.86–2.02)
								RDW = 14.8–16, HR (m) = = 1.55 (1.02–2.37)
								RDW > 16, HR (m) = 2.72 (1.83–4.05)
Cauthen CA	2012	USA	HF	ACM	6052	Age: 65 ± 14, 64.6% male	4.4 ± 2.4 y	HR (a) = 1.09 (1.01–1.17)*
Dabbah S	2010	Israel	MI	ACM	1709	Age: 61 ± 12, 78.2% male	27 m	RDW ≤ 12.8, HR (a) = 1
								RDW = 12.9–13.2, HR ((a) = 1.1 (0.6–2.1)
								RDW = 13.3–13.7, HR (a)1.8 (1–3.2)
								RDW = 13.8–14.3, HR (a) = 2 (1.1–3.4)
								RDW ≥ 14.4, HR (a) = 2.8 (1.6–4.7)
Ephrem G	2013	US	MI	MACE	543	Age: 65 ± 13, 56% male	3.8 y	HR (m) = 1.35 (1.02–1.79)*
Felker GM (1) CHARM Cohort	2007	USA	HF	ACM/MACE	2679	Age: 64.1 ± 11.5, 66.6% male	34 m	RDW ≤ 13.3, HR (a) = 1
								RDW = 13.3–14.0, HR (a) = 1.2 (1–1.7)
								RDW = 14.0–14.7, HR (a) = 1.1 (0.9–1.6)
								RDW = 14.7–15.8, HR (a) = 1.5 (1.1–2)
								RDW > 15.8, HR (a) = 1.7 (1.3–2.3)
								RDW<13.3 = 1
								RDW = 13.3–14, HR (m) = 1.1 (0.8–1.5)
								RDW = 14–14.7, HR (m) = 1.2 (1–1.6)
								RDW = 14.7–15.8, HR (m) = 1.5 (1.2–2)
								RDW > 15.8, HR (m) = 1.9 (1.5–2.4)
Felker GM (2) Duke Databank	2007	USA	HF	ACM	2140	NA	4y	RDW ≤ 13.0, HR (a) = 1
								RDW = 13.1–13.6, HR (a) = 1.6 (1.1–2.5)
								RDW = 13.7–14.2, HR (a) = 1.2 (0.8–1.8)
								RDW = 14.3–15.3, HR (a) = 1.5 (1–2.2)
								RDW > 15.3, HR (a) = 2.2 (1.5–3.3)
Gijsberts	2015	Netherla nd	MI	ACM/MACE	1760	Age: 66.2 ± 10.9, 72.7% male	42 m	HR (a) = 1.25 (1.04–1.49)*
								HR (m) = 1.19 (1.08–1.32)*
Jackson CE	2009	UK	HF	ACM	707	Age: 73 (67–80), 52% male	421 d	HR (a) = 1.06 (1.01–1.11)*
Jung C	2011	Geramny	HF	MACE	354	Age: 49 (median), 75.7% male	2579 d	HR (m) = 1.527 (1.274–1.831)*
Lappe J (a)	2013	USA	HF	ACM/MACE	6616	Age: 71.4 ± 14.6, 37.7% male	1 m	HR (a) = 1.111 (1.06–1.16)*
								HR (m) = 1.047 (1.01–1.08)*
Lappe JM (b)	2011	USA	CAD	ACM	1489	Age: 65.5 ± 11.3, 74.4% male	8.4–15.2 y	RDW<12.5, HR (a) = 1
								RDW = 12.5–12.8, HR (a) = 1.46 (1.05–1.86)
								RDW = 12.9–13.3, HR (a) = 1.64 (1.3–1.98)
								RDW = 13.4–14.2, HR (a) = 1.71 (1.53–1.88)
								RDW > 14.2, HR (a) = 3.02 (2.12–3.92)
Lee JH	2013	Korea	MI	MACE	1596	Age: 64.5 ± 11.9, 67.0% male	12 m	HR (m) = 1.19 (1.03–1.37)*
								RDW<12.6, HR (m) = 1
								RDW = 12.7–13.1, HR (m) = 4.24 (1.41–12.75)
								RDW = 13.2–13.9, HR (m) = 4.36 (1.47–12.91)
								RDW > 13.9, HR (m) = 6.18 (2.1–18.21)
Makhoul BF	2013	Israel	HF	ACM	614	Age: 77 ± 10, 45.9% male	1 y	HR (a) = 1.15 (1.08–1.21)*
								RDW ≤ 14.3, HR (a) = 1
								RDW = 14.4–15.2, HR (a) = 0.9 (0.6–1.3)
								RDW = 15.3–16.5, HR (a) = 1.2 (0.8–1.7)
								RDW ≥ 16.6, HR (a) = 1.9 (1.3–2.6)
Oh J	2012	Korea	HF	MACE	261	Age: 62.6 ± 14.2, 54.8% male	1 m	HR (m) = 1.14 (1.042–1.247)*
Osadnik T	2013	Poland	CAD	ACM	2550	Age: 64.4 ± 9.3, 70.5% male	2.5 y	HR (a) = 1.23 (1.13–1.35)*
Pascual-Figal DA	2009	Spain	HF	ACM	628	Age: 71 (61–77), 68% male	38.1 m	HR (a) = 1.074 (1.021–1.127)*
Poludasu S	2009	USA	CAD	ACM	859	Age: 62.3 ± 10.3, 49.4% male	4 y	RDW<13.3, HR (a) = 1
								RDW = 13.3–15.7, HR (a) = 0.91 (0.41–2)
								RDW ≥ 15.7, HR (a) = 3.48 (1.36–8.9)
Rickard J	2012	USA	HF	ACM	217	Age: 64.1 ± 11.8, 73.3% male	4.4 ± 1.8 y	HR (a) = 1.19 (1.06–1.33)
								RDW<13.6, HR (a) = 1
								RDW = 13.6–16.1, HR (a) = 1.19 (1.06–1.33)
								RDW ≥ 16.1, HR (a) = 2.49 (1.13–5.44)
Sahin O	2014	Turkey	MI	ACM	335	Age: 63 ± 13, 66.0% male	18 m	HR (a) = 0.97 (0.83–1.12)*
Tonelli M	2008	CARE study	CAD	ACM/MACE	4111	Age: 57.9 ± 9.2, 86.4% male	59.7 m	HR (a) = 1.14 (1.05–1.24)*
								HR (m) = 1.08 (1.00–1.17)*
								RDW ≤ 12.6, HR (a) = 1
								RDW = 12.6–13.1, HR (a) = 1.29 (0.92–1.82)
								RDW = 13.1–13.7, HR (a) = 1.35 (0.97–1.88)
								RDW ≥ 13.7, HR (a) = 1.78 (1.28–2.47)
								RDW = 10.9–12.6, HR (m) = 1
								RDW = 12.6–13.1, HR (m) = 1.19 (0.9–1.59)
								RDW = 13.1–13.7, HR (m) = 1.39 (1.05–1.83)
								RDW = 13.8–23.2, HR (m) = 1.56 (1.17–2.08)
Tseliou E	2014	Greece	HF	ACM/MACE	80	Age: 57.8 ± 12.4, 97.6% male	6 m	HR (a) = 1.15 (1.05–1.27)*
								HR (m) = 1.15 (1.05–1.27)*
Uyarel H	2011	Turkey	MI	MACE	2506	Age: 56.6 ± 11.8, 82.8% male	21 m	RDW<14.8, HR (m) = 1
								RDW > 14.8, HR (m) = 1.831 (1.034–3.24)
van Kimmenade	2009	USA	HF	ACM	205	Age: 73.1 ± 13, 51.2% male	1 y	HR (a) = 1.03 (1.02–1.07)*
Yao HM	2014	China	CAD	ACM/MACE	2169	Age: 60.2 ± 10.9, 67.7% male	2 y	HR (a) = 1.37 (1.15–1.62)*
								HR (m) = 1.21 (1.04–1.39)*
Yu SB	2012	China	HF	ACM	16681	Age: 66 (54–74), 49.3% male	3 y	RDW ≤ 13.2, HR (a) = 1
								RDW = 13.3–14.1, HR (a) = 0.892 (0.818–0.973)
								RDW = 14.2–14.8, HR (a) = 0.859 (0.793–0.931)
								RDW ≥ 14.9, HR (a) = 1.034 (0.961–1.111)
Zalawadiya SK	2011	USA	HF	ACM	789	Age: 62.7 ± 15.1, 50% male	573 d	HR (a) = 1.20 (1.11–1.29)*
								RDW ≤ 14, HR (a) = 1
								RDW = 14.01–15.20, HR (a) = 1.73 (0.94–3.19)
								RDW = 15.21–16.50, HR (a) = 2.44 (1.34–4.47)
								RDW > 16.5, HR (a) = 3.21 (1.77–5.83)

^*^Analysis for risk of per 1% increase of RDW; HR (a): hazard ratio for ACM; HR (m): hazard ratio for MACE; ACM: all-cause mortality; MACE: major adverse cardiac event; HF: heart failure; MI: myocardial infarction; CAD: coronary artery disease; 95% CI: 95% confidence intervals; d: days; m: months; y: years.
