# Supplementary material for: An overall and dose-response meta-analysis of red blood cell distribution width and CVD outcomes
Source: Sci Rep. 2017 Feb 24;7:43420. doi: 10.1038/srep43420 (PMC5324076; doi:10.1038/srep43420)
Supplement: Supplementary Materials [file srep43420-s1.pdf]

[Supplementary materials]

An overall and dose-response meta-analysis of red blood cell distribution width and CVD outcomes

Haifeng Hou[1,2]*, Tao Sun [1], Cheng Li [3], Yuanmin Li [4], Zheng Guo[1], Wei Wang[1,2], Dong Li [1]*

Supplementary Figures

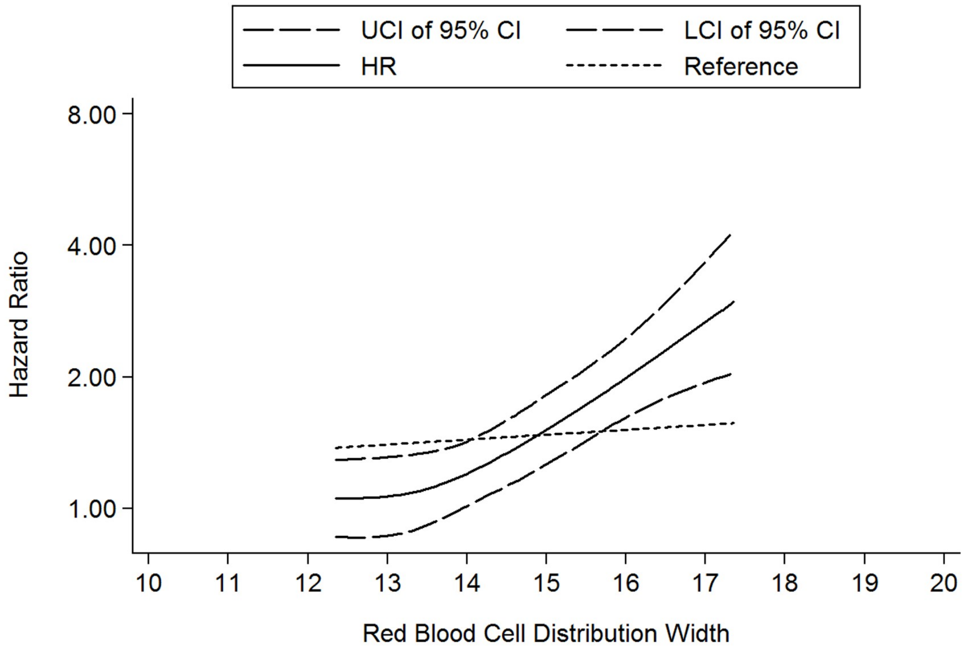

Supplementary Figure 1 Sensitivity analysis of the association between per unit of RDW increase and the risk of all-cause mortality in dose-response analysis

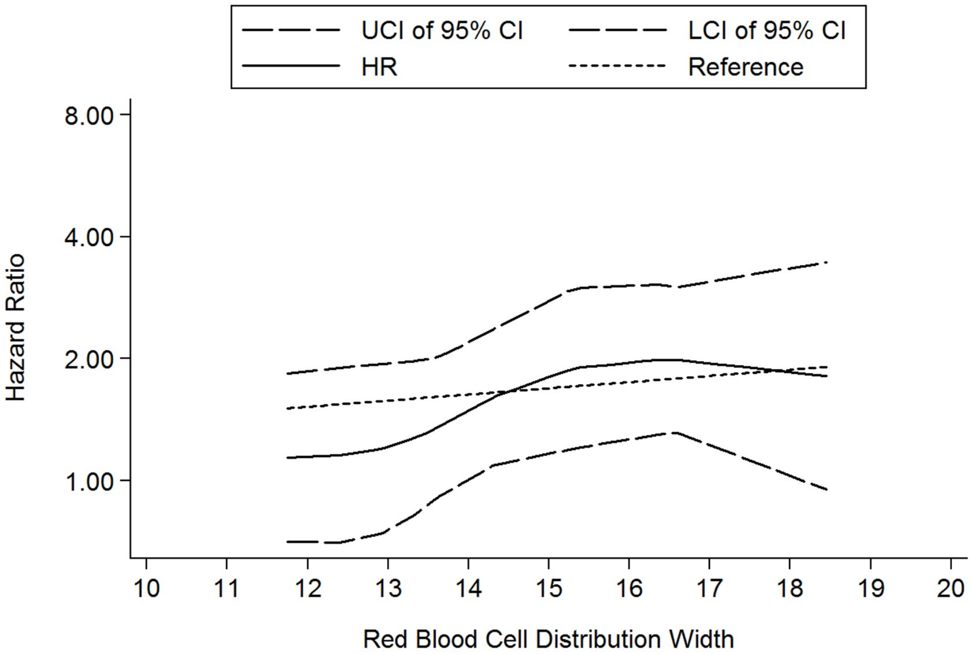

Supplementary Figure 2 Sensitivity analysis of the association between per unit of RDW increase and adverse cardiovascular events in dose-response analysis

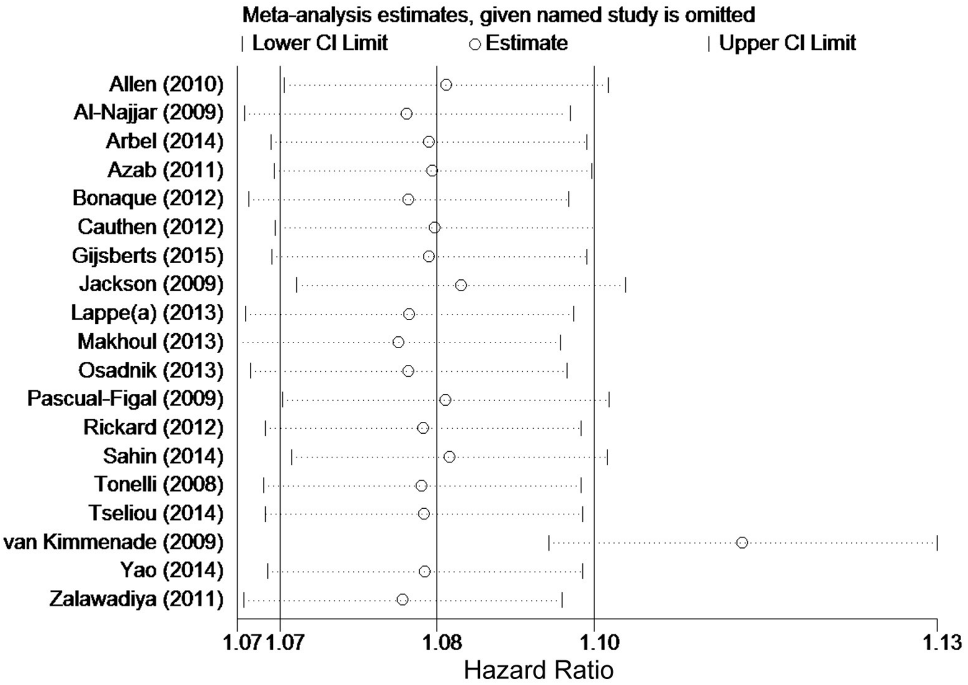

Supplementary Figure 3 Sensitivity analysis of the association between per 1% RDW increase and the risk of all-cause mortality in overall meta-analysis

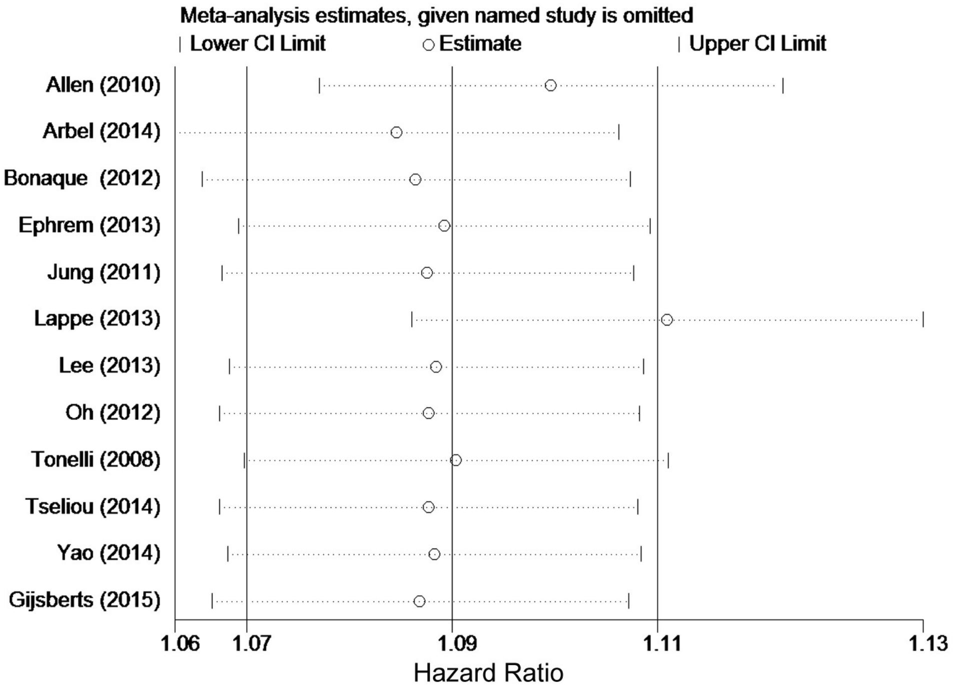

Supplementary Figure 4 Sensitivity analysis of the association between per 1% RDW increase and adverse cardiovascular events in overall meta-analysis

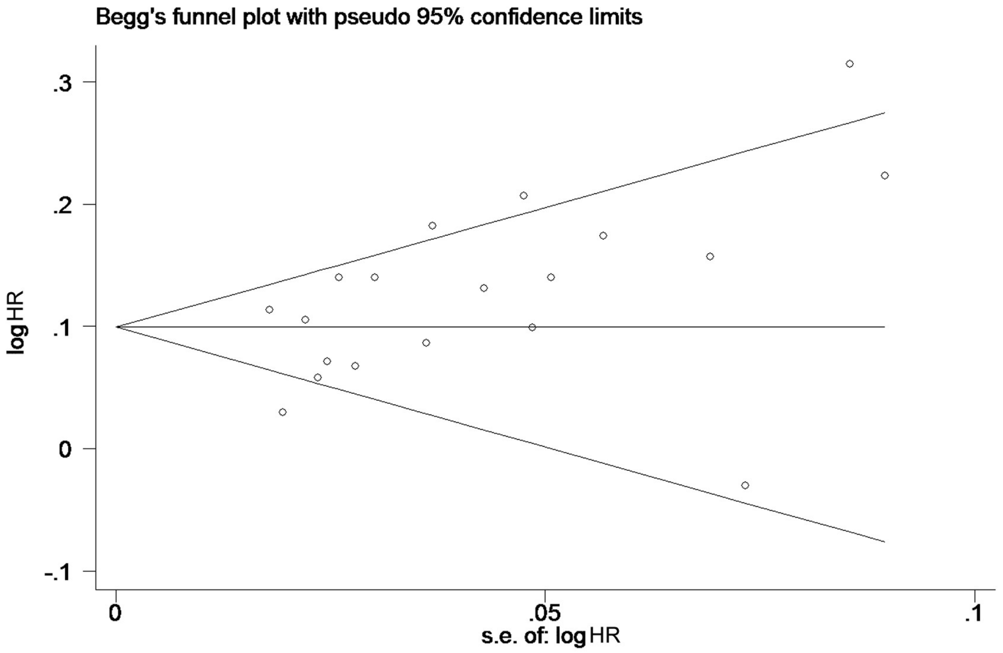

Supplementary Figure 5 Funnel plots of the association of per 1% RDW increase and the risk of all-cause mortality in overall meta-analysis. HR: hazard ratio

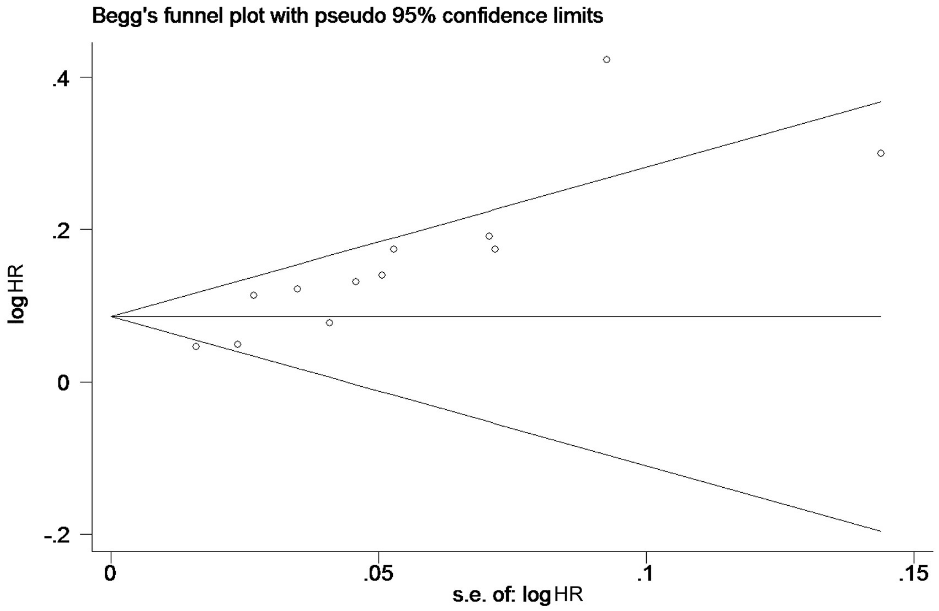

Supplementary Figure 6 Funnel plots of the association of per 1% RDW increase and adverse cardiovascular events in overall meta-analysis. HR: hazard ratio

Supplementary Tables

Supplementary Table 1 Preferred Reporting Items for Systematic Reviews and Meta-Analyses: The PRISMA Statement

Section/Topic	#	Checklist Item	Reported or not
**TITLE**			
Title	1	Identify the report as a systematic review, meta-analysis, or both.	Y
**ABSTRACT**			
Structured summary	2	Provide a structured summary including, as applicable: background; objectives; data sources; study eligibility criteria, participants, and interventions; study appraisal and synthesis methods; results; limitations; conclusions and implications of key findings; systematic review registration number.	Y
**INTRODUCTION**			
Rationale	3	Describe the rationale for the review in the context of what is already known.	Y
Objectives	4	Provide an explicit statement of questions being addressed with reference to participants, interventions, comparisons, outcomes, and study design (PICOS).	Y
**METHODS**			
Protocol and registration	5	Indicate if a review protocol exists, if and where it can be accessed (e.g., Web address), and, if available, provide registration information including registration number.	N
Eligibility criteria	6	Specify study characteristics (e.g., PICOS, length of follow-up) and report characteristics (e.g., years considered, language, publication status) used as criteria for eligibility, giving rationale.	Y
Information sources	7	Describe all information sources (e.g., databases with dates of coverage, contact with study authors to identify additional studies) in the search and date last searched.	Y
Search	8	Present full electronic search strategy for at least one database, including any limits used, such that it could be repeated.	Y
Study selection	9	State the process for selecting studies (i.e., screening, eligibility, included in systematic review, and, if applicable, included in the meta-analysis).	Y
Data collection process	10	Describe method of data extraction from reports (e.g., piloted forms, independently, in duplicate) and any processes for obtaining and confirming data from investigators.	Y
Data items	11	List and define all variables for which data were sought (e.g., PICOS, funding sources) and any assumptions and simplifications made.	Y
Risk of bias in individual studies	12	Describe methods used for assessing risk of bias of individual studies (including specification of whether this was done at the study or outcome level), and how this information is to be used in any data synthesis.	Y
Summary measures	13	State the principal summary measures (e.g., risk ratio, difference in means).	Y
Synthesis of results	14	Describe the methods of handling data and combining results of studies, if done, including measures of consistency (e.g., I^2) for each	Y

		meta-analysis.	
Risk of bias across studies	15	Specify any assessment of risk of bias that may affect the cumulative evidence (e.g., publication bias, selective reporting within studies).	Y
Additional analyses	16	Describe methods of additional analyses (e.g., sensitivity or subgroup analyses, meta-regression), if done, indicating which were pre-specified.	Y
**RESULTS**			
Study selection	17	Give numbers of studies screened, assessed for eligibility, and included in the review, with reasons for exclusions at each stage, ideally with a flow diagram.	Y
Study characteristics	18	For each study, present characteristics for which data were extracted (e.g., study size, PICOS, follow-up period) and provide the citations.	Y
Risk of bias within studies	19	Present data on risk of bias of each study and, if available, any outcome-level assessment (see Item 12).	Y
Results of individual studies	20	For all outcomes considered (benefits or harms), present, for each study: (a) simple summary data for each intervention group and (b) effect estimates and confidence intervals, ideally with a forest plot.	Y
Synthesis of results	21	Present results of each meta-analysis done, including confidence intervals and measures of consistency.	Y
Risk of bias across studies	22	Present results of any assessment of risk of bias across studies (see Item 15).	Y
Additional analysis	23	Give results of additional analyses, if done (e.g., sensitivity or subgroup analyses, meta-regression [see Item 16]).	Y
**DISCUSSION**			Y
Summary of evidence	24	Summarize the main findings including the strength of evidence for each main outcome; consider their relevance to key groups (e.g., health care providers, users, and policy makers).	Y
Limitations	25	Discuss limitations at study and outcome level (e.g., risk of bias), and at review level (e.g., incomplete retrieval of identified research, reporting bias).	Y
Conclusions	26	Provide a general interpretation of the results in the context of other evidence, and implications for future research.	Y
**FUNDING**			
Funding	27	Describe sources of funding for the systematic review and other support (e.g., supply of data)	Y

Y: the item was reported in article, N: the item was not reported.

Supplementary Table 2 Scale for quality assessment based on PRISMA statement and MOOSE guideline

Criteria	Score
**Representativeness of cases**	
Characteristics of participants were described.	1
Consecutive/randomly selected from case population was clearly defined.	1
Eligible patients are similar to controls, in term of age, gender and other important characteristics.	1
The percentage of loss to follow-up was provided, or the reasons of loss to follow-up were mentioned.	1
**Accuracy of information**	
Methods of variable measurement were offered.	1
Definitions of outcome were offered.	1
**Statistical analyses**	
Methods of statistical analyses were adequate to resolve research hypothesis.	1
Multivariate analyses were performed.	1
**Final question**	
If there were any other important flaws in the design, the study would be not included.	

Supplementary Table 3 Subgroup analysis for HR of per 1% RDW increase with all-cause mortality

Variable		Meta-analysis			Heterogeneity test			Model
		Pooled HR	95%CI	p	I^2(%)	Q	p	
Ethnicity								
	Caucasian	1.113	1.083-1.143	0.000	67.8	52.84	0.000	Random
	Asian	1.370	1.150-1.620	0.000	-	-	-	-
Design								
	Prospective	1.108	1.072-1.144	0.000	71.2	41.72	0.000	Random
	Retrospective	1.143	1.082-1.205	0.000	57.5	11.78	0.038	Random
Disease								
	CAD	1.211	1.107-1.315	0.000	47.5	3.81	0.149	Fixed
	MI	1.107	1.004-1.209	0.000	46.0	5.56	0.135	Fixed
	HF	1.105	1.073-1.138	0.000	71.3	38.35	0.000	Random
Follow-up Duration								
	<2years	1.093	1.049-1.137	0.000	76.7	30.00	0.000	Random
	≥2years	1.135	1.100-1.171	0.000	33.6	15.07	0.130	Fixed

HF: heart failure; MI: myocardial infarction; CAD: coronary artery disease; 95% CI: 95% confidence interval

Supplementary Table 4 Subgroup analysis for adjusted HR of per 1% RDW increase with major adverse cardiac events

Varible		Meta-analysis			Heterogeneity test			Model
		Pooled HR	95%CI	p	I^2(%)	Q	p	
Ethnicity								
	Caucasion	1.113	1.066-1.241	0.000	68.5	25.43	0.001	Random
	Asian	1.165	1.086-1.243	0.000	0	0.57	0.754	Fixed
Design								
	Prospective	1.142	1.079-1.206	0.000	73.5	6.99	0.001	Random
	Retrospective	1.100	1.039-1.161	0.000	42.7	22.61	0.137	Fixed
Disease								
	CAD	1.122	1.081-1.165	0.000	41.7	1.72	0.190	Fixed
	MI	1.140	1.080-1.209	0.000	0	2.65	0.449	Fixed
	HF	1.110	1.051-1.169	0.000	38.35	19.04	0.002	Random
Follow-up Duration								
	<2years	1.076	1.029-1.124	0.000	46.5	7.48	0.113	Fixed
	≥2years	1.147	1.095-1.200	0.000	46.0	11.10	0.085	Fixed

HF: heart failure; MI: myocardial infarction; CAD: coronary artery disease; 95% CI: 95% confidence interval
